# The molecular mechanisms of ferroptosis and its role in glioma progression and treatment

**DOI:** 10.3389/fonc.2022.917537

**Published:** 2022-08-16

**Authors:** Mengyang Lu, Yuanshuai Zhou, Linjuan Sun, Shaheryar Shafi, Nafees Ahmad, Minxuan Sun, Jun Dong

**Affiliations:** ^1^ Noncoding RNA and Cancer Lab, Faculty of Life Sciences, Shanghai University, Shanghai, China; ^2^ Jiangsu Key Laboratory of Medical Optics, Suzhou Institute of Biomedical Engineering and Technology, Chinese Academy of Sciences, Suzhou, China; ^3^ School of Biomedical Engineering, Division of Life Sciences and Medicine, University of Science and Technology of China, Hefei, China; ^4^ Institute of Biomedical and Genetic Engineering, Islamabad, Pakistan; ^5^ Department of Neurosurgery, The Second Affiliated Hospital of Soochow University, Suzhou, China

**Keywords:** ferroptosis, molecular mechanism, role, glioma progression, combination therapy

## Abstract

Ferroptosis is one of the programmed modes of cell death that has attracted widespread attention recently and is capable of influencing the developmental course and prognosis of many tumors. Glioma is one of the most common primary tumors of the central nervous system, but effective treatment options are very limited. Ferroptosis plays a critical role in the glioma progression, affecting tumor cell proliferation, angiogenesis, tumor necrosis, and shaping the immune-resistant tumor microenvironment. Inducing ferroptosis has emerged as an attractive strategy for glioma. In this paper, we review ferroptosis-related researches on glioma progression and treatment.

## Introduction

Ferroptosis is an iron-dependent form of programmed cell death, is more immunogenic than apoptosis. During ferroptosis, the level of reactive oxygen species (ROS) increases and induces lipid peroxidation (LPO) (1, 2). Ferroptosis is widely present in the development of many cancers, such as liver cancer, gastric cancer, lung cancer, colorectal cancer, ovarian cancer, breast cancer, glioma, and hematologic tumors (3). Ferroptosis has attracted increasing attention since its naming in 2012 (4).

The process of ferroptosis involves multiple signaling pathways and regulatory mechanisms that interact with other cell death modalities in the development of glioma (3, 5–7). It has been shown that increased ROS during ferroptosis can initiate LPO by interacting with polyunsaturated fatty acids in lipid membranes, thereby mediating chemoresistance in gliomas (8). A deeper understanding of the mechanism of ferroptosis in glioma progression is of great significance for the research and improving the existing therapies for glioma.

Taking into account the histopathological manifestations and alterations in genes, molecules and signaling pathways, in 2021, WHO proposed the fifth edition of the CNS tumor classification, which comprehensively introduced the latest classification criteria for gliomas, using the terms “diffuse” and “restrictive” to define different types of gliomas, replacing the original Roman numeral grading method of grade I-IV. The latest published classification shows that diffuse gliomas occurring mainly in adults and mainly in children have some molecular differences and should be classified as adult and pediatric types; moreover, adult diffuse gliomas that occur as angiodysplasia and necrosis should be diagnosed as glioblastoma (9).

**Figure 1:**
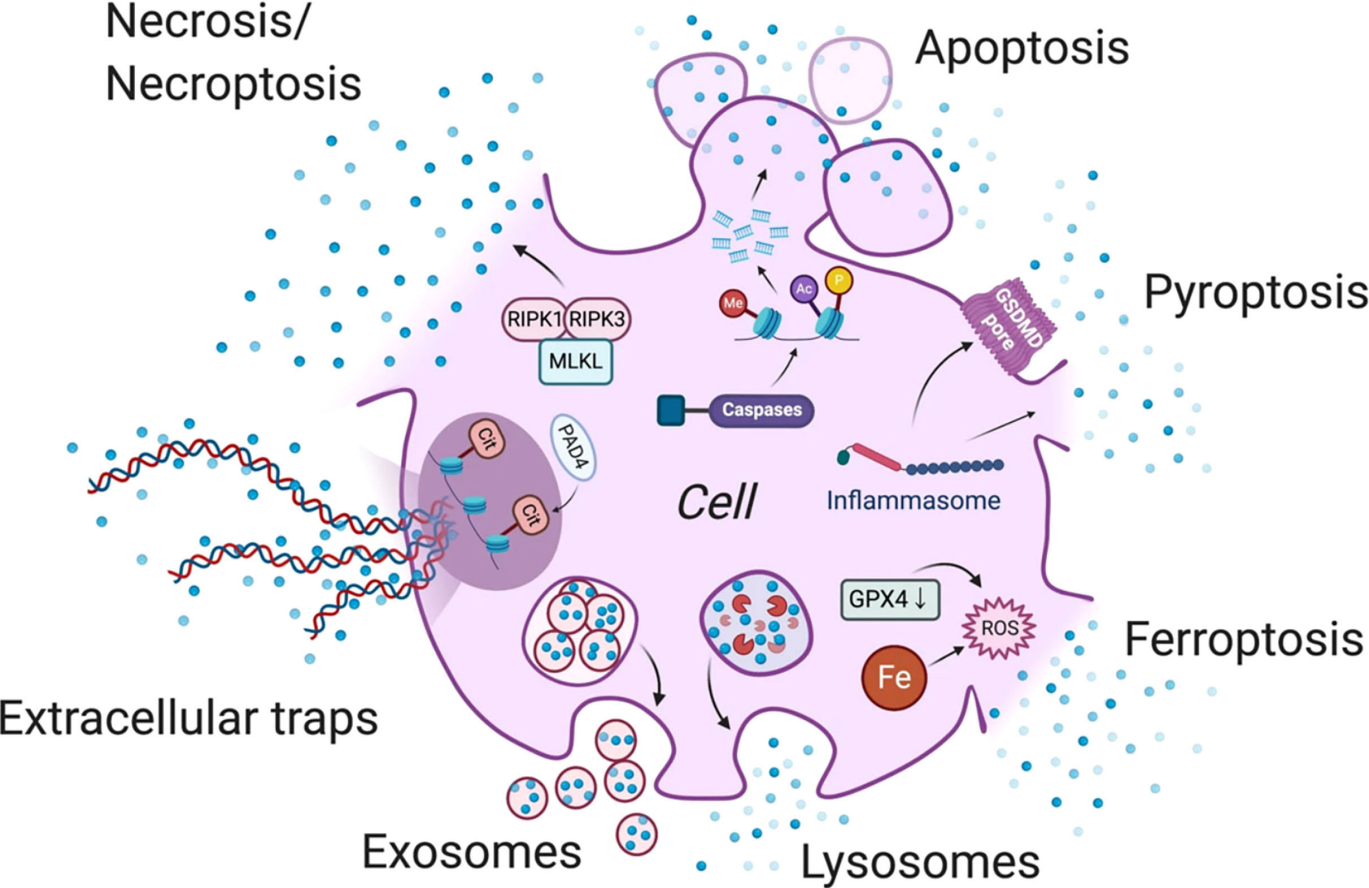
Universal mechanisms of DAMP release.

The damage-associated molecular pattern (DAMP) of ferroptosis is more specific than the other forms of cell death (**Figure 1**, Universal mechanisms of DAMP release) (10). On the one hand, ferroptosis can recruit and activate numerous immune cells at the tumor site and drive dendritic cell maturation *in vitro (* 11, 12), and ferroptosis inducers can function as sensitizers for anti-tumor immunotherapy (13–15). Studies have shown that ferroptosis combined with radiotherapy and chemotherapy can partially overcome drug resistance, limit glioma growth and prolong survival (14, 16, 17). Alternatively, ferroptosis is a unique form of autophagy (18), and results in iron accumulation, which is not only associated with iron uptake and new blood vessels formation during tumor growth (19), but also serves as an important factor involved in the construction of an immunosuppressive glioma microenvironment, such as the regulation of proliferation of B cells, T cells and immunophenotypic differentiation of tumor-associated macrophages (20). In conclusion, ferroptosis is involved in multiple aspects of the glioma progression, thus, targeting ferroptosis may be a potential strategy for glioma therapy.

## Molecular mechanism of ferroptosis

**Figure 2:**
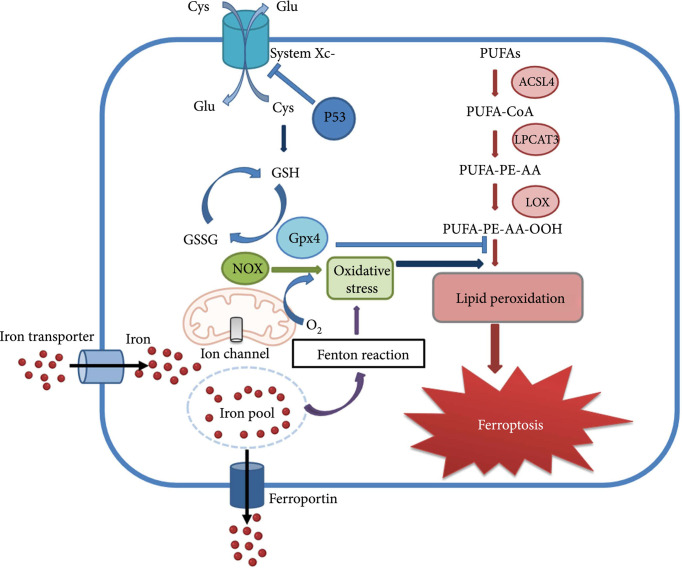
Molecular mechanism of ferroptosis.

In 2012, a new type of iron-dependent programmed cell death has been described and named ferroptosis by Professor Stockwell et al. (4). The process of ferroptosis can be briefly described as the activation of lipoxygenase by free ferrous ions through the Fenton reaction, leading to peroxidation of polyunsaturated fatty acids (PUFAs) on cell membranes, and the increased level of LPO causes loss of cell permeability and eventually cellular ferroptosis (21). Ferroptosis is regulated by a combination of iron metabolism, LPO and antioxidant systems, impairing the homeostasis of any of these processes may trigger ferroptosis (22). There are diverse cellular defense systems in response to LPO in cells, including the classical pathway mediated by GPX4, the non-classical pathway mediated by FSP1 independently of GPX4, as well as a third pathway in which dihydroorotate dehydrogenase (DHODH) interacts with GPX4 to block ferroptosis in the inner mitochondrial membrane by reducing ubiquinone to form ubiquinol (23). The ferroptosis related mechanism will be discussed in detail below (**Figure 2**, Molecular mechanism of ferroptosis) (24).

### The involvement of important molecules in the process of ferroptosis

Glutathione peroxidase 4(GPX4), is a crucial regulator of endogenous ferroptosis. GPX4 can convert glutathione (GSH) to oxidized glutathione and reduce cytotoxic lipid peroxides to the corresponding alcohols. By inhibiting the formation of lipid peroxides, GPX4 prevents the production of LPO reaction products, reduces cell membrane damage, and thus alleviates cellular ferroptosis (25). Down-regulation of GPX4 expression can make cells more sensitive to ferroptosis, and knockdown of GPX4 induces ferroptosis. Whereas studies show that GPX4 expression was significantly increased in human glioma patients compared to brain tissue of healthy patients (26).

The X^c-^system, a heterodimer composed of subunits SLC7A11 and SLC3A2, is an important component of the cellular antioxidant system and is closely associated with exogenous ferroptosis processes. It is widely distributed in the phospholipid bilayer and is involved in intracellular cystine uptake and GSH synthesis (27). The inhibition activity of the X^c-^system will result in reduced GPX4 activity, ROS accumulation, and lipid oxidative stress, causing cellular ferroptosis. SLC7A11 was reported to be expressed at higher levels in GBM patient biopsies or glioma cell lines than in normal brain tissue (28).

ACSL4, acyl-CoA long-chain synthase 4, expressed in the endoplasmic reticulum and mitochondrial outer membrane, is an essential molecule in lipid metabolism. This molecule is mainly responsible for catalyzing the formation of acetyl coenzyme A from lipids. It is closely related to the production of ROS and the process of ferroptosis, and thus has potential to be an indicator of ferroptosis sensitivity (29). ACSL4 down-regulates glioma cell proliferation and mediates up-regulation of ferroptosis levels in gliomas. Studies have shown that ACSL4 expression is down-regulated after glioma occurs (30). Additionally, it was reported that GPX4 knockdown leads to ferroptosis, while double knockdown of GPX4 and ACSL4 genes can reverse GPX4 knockdown-induced ferroptosis (31).

FSP1 (ferroptosis-suppressor-protein1), is a ubiquinone oxidoreductase, it was initially described as a pro-apoptotic gene called apoptosis-inducing factor 2(AIFM2) in mitochondria and is now considered as a glutathione-independent ferroptosis resistance molecule. FSP1 can act in parallel with the GPX4 pathway, thus preventing glutathione deficiency-induced ferroptosis (32).

Moreover, FSP1 can use NADPH to catalyze the reduction of the lipophilic radical scavenger ubiquinone (CoQ10), and the FSP1-CoQ10-NADPH pathway can synergistically inhibit ROS elevation with the GPX4 system to prevent ferroptosis caused by oxidative damage (33).

DHODH, an iron-containing flavin-dependent enzyme, is an important molecule in nucleotide metabolism, which inhibits mitochondrial ferroptosis *via* regulating the production of the antioxidant ubiquinol(CoQH2) in the inner mitochondrial membrane (34). It was shown that inhibition of DHODH promotes ferroptosis as it increases LPO in mitochondria, and DHODH can act synergistically with GPX4 to inhibit ferroptosis in the mitochondrial inner membrane (35).

*TP53*, a widely studied oncogene, can repress the expression of SLC7A11, a component of the X^c-^system, at the transcriptional level, in turn targeting the diamine acetyltransferase SAT1 and the mitochondrial glutaminase GLS2, which are involved in the regulation of glutamine metabolism, to enhance cellular ferroptosis (36).

Furthermore, p53 can inhibit ferroptosis by directly inhibiting dipeptidyl peptidase 4 (DPP4) activity or by inducing cell cycle protein-dependent kinase inhibitor 1A (CDKN1A/p21) expression (36). Recent studies have shown that the regulation of ferroptosis by p53 contributes to its tumor suppressive function (37). In addition, p53 plays a dual role in mediating ferroptosis in glioma (38).

Nrf2, a transcription factor that regulates redox metabolism, contains a basic leucine zipper DNA-binding domain at the C-terminus and plays a key role in the cellular response to oxidative stress. Many enzymes and proteins involved in LPO are target genes of NRF2, such as glutamate-cysteine ligase and glutathione peroxidase (GPX). Through interactions with p53, GPX4, X^c-^system, etc., Nrf2 affects ferroptosis (39).

## The role of ferroptosis in glioma progression

The relevance of ferroptosis and glioma has been widely recognized for influencing various vital processes in the development of glioma. It is not only involved in the construction of an acidic, hypoxic, immunosuppressive glioma microenvironment, but is also closely related to glioma cell proliferation, angiogenesis, tumor necrosis, and invasive growth.

Induced ferroptosis can mediate altered oxidative metabolism in glioma cells, trigger changes toward of macrophage polarization in the glioma microenvironment, and interfere with the proliferation and function of immune cells.

### Ferroptosis influence glioma cell proliferation

Ferroptosis has been proposed to play an important role in glioma cell proliferation (40, 41). Inhibition of ferroptosis accelerates glioma proliferation and metastasis and promotes angiogenesis and malignant transformation of gliomas. Ferroptosis attenuates the viability of glioma cells, and activation of ferroptosis inhibits glioma cell proliferation. It was revealed that reduced ferroptosis in human glioma tissue and glioma cells might be associated with ACSL4, an important molecule in the emergence of ferroptosis (30). This study found ACSL4 expression was decreased in glioma cells and reduced expression of ACSL4 compared to the normal human brain. Furthermore, their speculates confirmed that ACSL4 plays an essential role in regulating ferroptosis and proliferation in glioma cells and knocking down the gene significantly improved the viability of glioma cells (30). Another investigation on non-coding RNA MicroRNA-670-3p again demonstrated that targeting the inhibition of ACSL4 and thus ferroptosis in human glioblastoma cells has a pro-tumor effect (42).

It was noted in studies on drugs that dihydrotanshinone I, a natural antitumor drug commonly used in clinical practice, can significantly proliferate human glioma cells and promote human glioma cell death (43). After treatment of human glioma cells with dihydrotanshinone I, GPX4 expression decreased while ACSL4 expression increased, inducing ferroptosis in human glioma cells. Then, the inhibitory effect of dihydrotanshinone I on the proliferation of glioma cells was blocked after the application of ferroptosis inhibitors (43). It can be concluded that the effect of dihydrotanshinone I on the proliferation of glioma cells is derived from ferroptosis. Additionally, elevated expression levels of Nrf2, a ferroptosis-related molecule, in glioma patient samples exhibited pro-tumor proliferative utility by regulating the X^c-^system-mediated reduction in ferroptosis (44).

### Ferroptosis promotes the progression of glioma necrosis

Tumor necrosis is a prevalent phenomenon in gliomas, especially high-grade glioblastomas, which is strongly associated with the highly aggressive growth of the tumor. Necrosis progresses alongside tumor progression, causing inflammation and cytokine storms resulting in multi-organ disorders, poor prognosis and death; the degree of tumor necrosis is negatively correlated with survival in glioma patients (45). There is a general consensus that tumor necrosis is caused by a hypoxic tumor microenvironment and rapidly proliferating tumor cells that exceed the capacity of the vascular supply (46). Moreover, it is proposed that necrosis is caused by iron-dependent oxidative stress and may partially follow the ferroptosis pattern (47). Glioma tissue that undergoes necrosis recruits immune cells by releasing its corresponding DAMP. Findings show that neutrophils extensively infiltrate the tumor necrosis area and increase with tumor progression, and that the degree of infiltration of glioma tumor-associated neutrophils positively correlates with the degree of tumor necrosis (47).

Furthermore, studies suggest that neutrophils are participating in the process of promoting tumor necrosis and that process is achieved by triggering ferroptosis in tumor cells. Besides, elevated glutamate levels in areas of glioma necrosis can cause increased tumor necrosis by inhibiting the X^c-^system in ferroptosis, thereby inducing ferroptosis (47).

### Ferroptosis has an impact on glioma angiogenesis

Aberrant vascular network formation with high permeability is another important feature and critical event in the glioma process. The formation of new blood vessels effectively promotes the infiltrative spread of gliomas, highly aggressive growth and leads to a therapeutic resistance. Augmented microvessels are mostly observed in areas of tumor infiltration and necrosis, and the density correlates positively with the malignancy of the glioma. It is believed that glioblastoma is one of the most vascularized human tumors (48). Glioma-associated macrophages, which play an invaluable role in iron metabolism and ferroptosis, are the most abundant immune cells in the glioma microenvironment and are engaged in all phases of angiogenesis, ranging from angiogenesis early sprouting to late neovascularization and the stabilization of neovascularization (49). It was reported that the number of macrophages around proliferating micro-vessels in glioblastoma was significantly increased. The release of angiogenic factors was promoted by stimulation of macrophages, while macrophages in gliomas promoted neovascularization through cyclooxygenase 2-mediated secretion of cyclooxygenase 2(COX2) and IL-6. Studies revealed that ferroptosis recruits glioma-associated macrophages and indirectly acts on macrophage-mediated neointima formation (50). The other evidence that ferroptosis influences glioma angiogenesis has been derived from studies of the transcriptional activator ATF4. It is an integral molecule in cellular oxidative metabolism and is highly expressed in gliomas, promoting cell migration and anchorage-independent cell growth, allowing tumor cells to adapt to the glioma microenvironment, thus, ATF4 plays a role in promoting proliferation and angiogenesis in gliomas (19). Furthermore, ATF4 acts in an X^c-^system-dependent manner, mediated by the SCL7A11 molecule. Findings show that the ferroptosis inducers erastin and RSL3 reduce ATF4-induced tumor angiogenesis (19).

### Ferroptosis raises immune resistance to glioma

Resistance to treatment in glioma is mainly due to the immunosuppressed glioma microenvironment, which is a major obstacle to glioma treatment. Ferroptosis has dual aspects in the glioma progression. On the one hand, ferroptosis is the main form of programmed cell death in the glioma process, causing tumor cell death; On the other hand, ferroptosis is engaged in shaping the immunosuppressive glioma microenvironment, contributing to a decrease in the host’s anti-tumor immunity and promoting tumor propagation (51). Bioinformatics data analysis revealed that the expression of ferroptosis-related genes was associated with immunosuppression in gliomas, and studies showed that the severity of ferroptosis was significantly associated with the clinical prognosis of gliomas (41). There is a large number of immune cells infiltrating the ferroptosis-enriched glioma. However, most of these immune cells are immunomodulatory cells, such as Treg, neutrophils, and glioma-associated macrophages. A subset of glioma-associated macrophages can be divided into two subtypes, M1 and M2, representing two different forms of effects that inhibit and promote tumor progression (52, 53). A study of 1750 patients showed that a higher proportion of tumor-promoting M2-type macrophages was demonstrated in the immunosuppressed glioma microenvironment. Furthermore, ferroptosis in gliomas promotes macrophage infiltration and induces M2-type polarization of macrophages (46). Being present in the vascular niches in close contact with brain endothelial cells, glioma stem cells have tumor initiation properties and self-renewal ability that contribute to the immunosuppressive microenvironment of glioma. These findings show that ferroptosis is involved in the stemness regulation of glioma stem cells. As an example, OTUB1, a deubiquitinating enzyme overexpressed in gliomas, regulates SLC7A11, a critical inhibitory molecule in the ferroptosis X^c-^system, directly through the ubiquitinase-proteasome degradation system, forming the OTUB1/SLC7A11 axis and thus promoting the stemness of glioma cells (54).

## Ferroptosis involvement in glioma treatment

A standard therapy for glioma treatment is a combination of surgery, radiotherapy, and chemotherapy, but the effectiveness of these therapies is limited due to the inherent treatment resistance of glioma. Being one of the important forms of cell death, ferroptosis can suppress the development of glioma. Studies have shown that molecules causing ferroptosis can play an aggressive role in the treatment of glioma (6, 55), and as such, ferroptosis can be used as a combination therapy in the treatment of glioma, improving the sensitivity of radiotherapy and chemotherapy. In conclusion, targeting ferroptosis-related genes might have potential value in the treatment of glioma.

### Inducing ferroptosis promotes sensitivity to glioma therapy

Radiotherapy (RT), an important component of standard therapy for glioma, uses X-rays to destroy tumor tissue and can directly trigger multiple types of DNA damage, such as base damage, single-strand breaks (SSB), double-strand breaks (DSB), thereby inducing cycle arrest, senescence, and multiple forms of death in highly proliferative tumor cells with some therapeutic effect. However, due to the heterogeneity of glioma, radiation therapy’s effect is inadequate (56). Studies suggest that the combination of ferroptosis inducer sorafenib and radiotherapy play a collaborative role in killing glioma cells (17). Alternatively, ferroptosis inducers 2-nitroimidazole, doranidazole and misonidazole can mediate altered oxidative stress metabolism in glioma stem cells, such as elevated levels of metal reductase steap3 and NADH by doranidazole, which can act as a sensitizer to counteract resistance to radiotherapy and produce cytotoxicity to limit glioma growth and significantly prolong survival (13) (more details see **Table 1**).

**Table 1:** This table lists compounds currently known to induce or promote ferroptosis as sensitizers in the treatment of glioma.

Classification	Compound	Mechanism
Class I ferroptosis inducers	Erastin	Inhibit SLC7A11 activity
	PE	Inhibit SLC7A11 activity
	IKE	Inhibit SLC7A11 activity
	SAS	Inhibit SLC7A11 activity
	Sorafenib	Inhibit SLC7A11 activity
	Glutamate	Inhibit SLC7A11 activity
	BSO	GSH depletion
	DPI2	GSH depletion
	Cisplatin	GSH depletion
Class II ferroptosis inducers	1S,3R-RSL3	Inhibit GPX4 activity
	ML162	Inhibit GPX4 activity
	ML210	Inhibit GPX4 activity
	Altretamine	Inhibit GPX4 activity
	Withaferin A	Inactivate/deplete GPX4
Class III ferroptosis inducers	FIN56	Degrade GPX4, activate SQS and deplete CoQ10
	Statins (fluvastatin, simvas-tatin, lovastatin acid)	Inhibit HMG-CoA reductase (inhibit CoQ10 synthesis, reduce GPX4 expression)
Class IV ferroptosis inducers	Ferric ammonium citrate/sulfate	Iron loading
	FeCl2	Iron loading
	Hemoglobin	Iron loading
	Hemin	Iron loading
	Nonthermal plasma	Promote the release of Fe2+ from ferritin
	Lapatinib + siramesine	Upregulate TfR1 and downregulate FPN1
	Salinomycin	Inhibit iron translocation and deplete ferritin
	Artesunate, DHA	Endogenous Fe2+ causes the cleavage of endoperoxide bridge
	FINO2	Inhibit GPX4 activity, Oxidize ferrous iron and lipidome
Other ferroptosis inducers and promoters	BAY 87–2243	Inhibit mitochondrial complex I
	BAY 11–7085	Upregulate HMOX1
	Auranofin/Ferroptocide	Inhibit thioredoxin
	iFSP1	Inhibit FSP1
	4-CBA	CoQ10 depletion
	DAHP	Inhibit GCH1
	Methotrexate	Inhibit DHFR
	MF-438/CAY10566	Inhibit SCD1
	JQ-1	Promote ferritinophagy

Oral alkylating agent TMZ is the first-line chemotherapeutic agent in the treatment of glioma. With the advantages of easy penetration of the blood-brain barrier, stable acidic environment and no superimposed toxicity with other drugs, it can prolong the survival time of glioma patients to some extent, but only partial patients can benefit from TMZ chemotherapy due to drug resistance (57). Several studies have found that the use of ferroptosis-inducing agents can increase TMZ sensitivity. The combined use of the ferroptosis inducer erastin and TMZ was reported to enhance TMZ sensitivity through multiple pathways (14); *in vitro* use of hydroxychloroquine (HCQ) and its derivative quinacrine(QN), which traverses the blood-brain barrier and impairs TMZ-induced autophagy, can induce ferroptosis and thus increase TMZ sensitivity (6) (more details see **Table 1**).

### Ferroptosis-related drugs in the treatment of glioma

Many drugs can work in glioma treatment by affecting the process of ferroptosis. Dihydroartemisinin (DHA) has been shown to exert anticancer activity by enhancing ferroptosis through the production of ROS and inhibition of GPX4 initiation (58). Amentoflavone (AF), a polyphenol widely found in cypress, has anti-inflammatory and anti-tumor effects. Findings show that AF can trigger glioma ferroptosis in an autophagy-dependent manner to exert anti-tumor effects (59). The accumulation of reactive oxygen species and LPO can be observed in glioma cells treated with the curcumin analogue ALZ003.*In vitro* and animal studies have shown that ALZ003 can inhibit the growth of TMZ-resistant gliomas by acting on GPX4, a crucial molecule in the ferroptosis pathway, while having no cytotoxic effect on normal astrocytes (15) (more details see **Table 1**).

Furthermore, due to the tight relationship between ferroptosis and lipid metabolism, many glioma therapeutic agents can exert therapeutic effects by mediating cellular ferroptosis through LPO. Brucine, an indole alkaloid extracted from the seeds of strychnine, promotes LPO, causing ferroptosis in glioma cells eventually inhibiting glioma cell growth *in vitro* and *in vivo (* 60). The non-steroidal anti-inflammatory drug (NSAID) ibuprofen induces ferroptosis of glioma cells, and its effects are coupled with an abnormal increase in intracellular LPO (61).

### Ferroptosis-based combination therapy in glioma

It was shown that NFKB activating protein(NKAP), an important regulator of mitosis, can positively regulate SLC7A11, a key molecule of ferroptosis, and knockdown of NKAP gene can increase the level of LPO and cause oxidative damage, which in turn induces glioma ferroptosis and suppresses glioma progression (62). Furthermore, the knockdown of NKAP gene resulted in glioma cell lines that were more sensitive to ferroptosis inducers. Based on this, we think that NKAP knockdown combined with ferroptosis induction therapy has the potential to be used in the treatment of glioma.

A homologous protein mouse double minute (MDM2) and murine double minute X (MDMX) form a complex that promotes ferroptosis sensitivity in glioma cells (63). The study revealed this complex inhibits cellular antioxidant defense by modulating the activity of the major lipid regulator PPARα, which influences cellular lipid metabolism and promotes oxidative damage, leading to LPO-mediated cellular ferroptosis (63). There is a potential to design combined ferroptosis-inducing therapy with MDM2 and MDMX agonists for application in the treatment of glioma.

It is believed that the increase in intracellular iron ions, which contributes to the increase in the unstable iron pool, promotes the Fenton reaction and the generation of toxic phospholipid hydrides can induce glioma ferroptosis. Study reveals that gallic acid (GA) can effectively reduce Fe^3+^ to Fe^2+^ and is able to induce ferroptosis in GBM cells as a substrate for the sustained Fenton reaction (64, 65). Zhang et al. designed a GA-based targeted nanomedicine that combines ferroptosis and photothermal therapy for glioma treatment (65) (more details see **Table 2**).

**Table 2:** This table lists nanoparticles currently known to induce or promote ferroptosis in the treatment of glioma.

Classification	Compound	Mechanism
Nanoparticles	AMSNs	GSH depletion
	LDL‐DHA	Loading natural omega 3 fatty acid
	ZVI NPs	Iron loadin
	FeGd-HN@Pt@LF/	Increase intracellular Fe2+ and H2O2 levels
	RGD2	delivery system releasing Fe3+ and doxorubici
	DGU : Fe/Dox	Consists of Fe3+ ion, tannic acid and sorafenib
	SRF@FeIIITA	Increase intracellular
	PSAF NCs	Fe2+ levels
	MON‐p53	Iron loading, inhibit SLC7A11

**Tables 1** and **2**, references from the article(Ferroptosis, radiotherapy, and combination therapeutic strategies).

## Conclusion and outlook

Glioma is the most common primary brain tumor in the central nervous system. The current standard treatment for glioma improves slightly the survival of patients, and all the treatments have shown some limitations and drug resistance. Ferroptosis is a newly defined form of cell death that plays an important role in the progression of glioma, affecting glioma cell proliferation, invasion, tumor necrosis, angiogenesis, and participating in the construction of an immunosuppressive glioma microenvironment. Moreover, ferroptosis can interfere with other modes of cell death. Thus, the induction of ferroptosis in gliomas has the potential to be a new option beyond standard therapies. Existing clinical reports and drug studies have shown that ferroptosis inducers used in combination with radiotherapy or TMZ can improve glioma treatment resistance, many drugs based on ferroptosis can play an aggressive role in glioma treatment, and targeting ferroptosis can contribute to the improvement of glioma treatment outcome. In order to apply ferroptosis in glioma treatment further, we need to perform a more in-depth study of the mechanisms involved in ferroptosis, to identify the population for which ferroptosis therapy is suitable; the toxicity of ferroptosis-inducing drugs, and drug delivery issues, we need to answer how to cross the blood-brain barrier effectively while avoiding off-target effects.

## Author contributions

ML wrote and revised the review article; YZ established the article outline and revised it; LS provided some of the material; SS and NA have adjusted some expressions of the article. All authors contributed to the article and approved the submitted version.

## Funding

This work was supported by Young Scientists Fund of the National Natural Science Foundation of China (81903028), China Postdoctoral Science Foundation (2020M671599), Jiangsu province key research and development program: Social development project(BE20211653), Key Projects of Jiangsu Provincial Health Care Commission Scientific Research Project (ZDB2020016), and Projects of International Cooperation of Jiangsu Province (No. BZ2020004).

## Conflict of interest

The authors declare that the research was conducted in the absence of any commercial or financial relationships that could be construed as a potential conflict of interest.

## Publisher’s Note

All claims expressed in this article are solely those of the authors and do not necessarily represent those of their affiliated organizations, or those of the publisher, the editors and the reviewers. Any product that may be evaluated in this article, or claim that may be made by its manufacturer, is not guaranteed or endorsed by the publisher.
